# Reference bias: presentation of extreme health states prior to eq-vas improves health-related quality of life scores. a randomised cross-over trial

**DOI:** 10.1186/1477-7525-8-146

**Published:** 2010-12-02

**Authors:** Steven McPhail, Elaine Beller, Terry Haines

**Affiliations:** 1 Centre for Functioning and Health Research, Ipswich Road, Woolloongabba, Queensland, Australia; 2 The University of Queensland, School of Health and Rehabilitation Sciences, St Lucia, Queensland, Australia; 3 Queensland University of Technology, School of Public Health and Institute of Health and Biomedical Innovation, Kelvin Grove, Australia; 4 Bond University, Centre for Research in Evidence-Based Practice, Gold Coast, Queensland, Australia; 5 Southern Health, Allied Health Research Unit, Kingston Centre, Cnr Warrigal and Kingston Roads, Cheltenham, Victoria, Australia; 6 Monash University, Physiotherapy Department, School of Primary Health Care, Monash University Peninsular Campus, Victoria, Australia

## Abstract

**Background:**

Clinical practice and clinical research has made a concerted effort to move beyond the use of clinical indicators alone and embrace patient focused care through the use of patient reported outcomes such as health-related quality of life. However, unless patients give consistent consideration to the health states that give meaning to measurement scales used to evaluate these constructs, longitudinal comparison of these measures may be invalid. This study aimed to investigate whether patients give consideration to a standard health state rating scale (EQ-VAS) and whether consideration of good and poor health state descriptors immediately changes their self-report.

**Methods:**

A randomised crossover trial was implemented amongst hospitalised older adults (n = 151). Patients were asked to consider descriptions of extremely good (Description-A) and poor (Description-B) health states. The EQ-VAS was administered as a self-report at baseline, after the first descriptors (A or B), then again after the remaining descriptors (B or A respectively). At baseline patients were also asked if they had considered either EQ-VAS anchors.

**Results:**

Overall 106/151 (70%) participants changed their self-evaluation by ≥5 points on the 100 point VAS, with a mean (SD) change of +4.5 (12) points (p < 0.001). A total of 74/151 (49%) participants did not consider the best health VAS anchor, of the 77 who did 59 (77%) thought the good health descriptors were more extreme (better) then they had previously considered. Similarly 85/151 (66%) participants did not consider the worst health anchor of the 66 who did 63 (95%) thought the poor health descriptors were more extreme (worse) then they had previously considered.

**Conclusions:**

Health state self-reports may not be well considered. An immediate significant shift in response can be elicited by exposure to a mere description of an extreme health state despite no actual change in underlying health state occurring. Caution should be exercised in research and clinical settings when interpreting subjective patient reported outcomes that are dependent on brief anchors for meaning.

**Trial Registration:**

Australian and New Zealand Clinical Trials Registry (#ACTRN12607000606482) http://www.anzctr.org.au

## Background

Over past decades, clinical practice and clinical research has made a concerted effort to move beyond the use of clinical indicators alone and embrace patient focused care[1]. Along this line, the evaluation of health-related quality of life (HRQoL) has great benefit in revealing how each patient views their own health state. Subjective HRQoL evaluation has particular importance amongst patient groups suffering from chronic, degenerative or terminal conditions where the aim of health interventions are to improve quality of life rather than for a curative effect[2,3]. It is not surprising then, that the use of generic HRQoL evaluation instruments, such as the Euroqol-5D (EQ-5D), have become increasingly popular as a primary outcome measure in clinical trials and as a primary instrument for economic evaluation through cost-utility analysis[4].

Concerns have been raised about the validity of making comparisons between HRQoL evaluations taken at different time points as change in ones understanding or perception of the HRQoL construct may occur between assessments [5-8]. If a respondent were to change their understanding of what components are included in the construct of HRQoL (reconceptualisation), or the relative importance of certain components of HRQoL in relation to the other components (reprioritisation) or change their internal perception of the relative value of certain health states in relation to others (recalibration), then each evaluation may not necessarily be measuring the same concept, with the same value system on the same scale despite consistent use of the same patient reported outcome [5-7]. This phenomenon has been given the term 'response shift.'

Response shift is generally considered to be part of naturally occurring adaptive processes and may help individuals adjust to living with poor health states and thus may be a desirable coping mechanism or even the goal of some treatments[6,7,9-11]. However, it also threatens to invalidate comparisons of pre and post intervention assessments or assessments taken over multiple time points in the trajectory of a chronic disease, despite use of a standardised instrument[6,7,9,11-13]. For this reason a number of methods to detect response shift, such as the 'then-test' (a retrospective report of a previous health state from the respondent's current perspective)[5,8,11,14,15] and 'structural equation modelling' (mathematical modelling to detect changes in factor solutions and variance-covariance matrices over time)[12,15,16] have been developed to evaluate response shift between assessments. However, these methods can often be time consuming, complex or burdensome on patients[5,7,11,15]. Detailed discussion of methods to detect response shift has previously been described[5,7,11,15,17].

It may not be possible (or desirable) to eliminate adaptive processes that contribute to response shift[5,7,11]. However, a potentially preventable (and undesirable) response shift artefact may occur as a result of subjective HRQoL appraisal processes. This may occur when a respondent does not give consistent consideration to questions used to evaluate their HRQoL at each assessment point. Subjective scales dependent on brief anchor descriptions to give meaning to the scale may be particularly prone to inconsistent consideration of the instrument, as a change in consideration of one or both anchors may lead to a substantial difference in response[11].

The EQ-VAS is the health state rating scale from the popular EQ-5D generic health-related quality of life instrument. The EQ-VAS includes a 100 point visual analogue rating scale with a bottom anchor of 'worst imaginable health' and a top anchor of 'best imaginable health'[18]. The EQ-VAS has favourable empirical evidence supporting its sensitivity to change, validity and reliability[19-27]. However, an investigation of EQ-VAS use in rating multiple hypothetical health states found that the rating given to common moderate health states were affected by the context in which they were presented[28]. It was noted that moderate health states were assigned lower values when presented in the context of more mild (better) health states and assigned higher values when presented in the context of more severe (worse) health states [28]. This is not an isolated finding for rating scales[29].

There is also evidence from other fields that framing a question to focus on positive or negative attributes can yield different responses despite no difference in logical meaning[30-33]. Empirical investigations of the framing effect generally suggest respondents demonstrate preference for an option with a positive valence rather than negative[31-33]. A simple example includes respondents reporting ground mince as 'tastier' when labelled as 75% lean, rather than 25% fat[34]. Framing effects have been applied in a wide range of fields including politics, consumer behaviour and health[30-34].

Respondents completing health state rating scales (like the EQ-VAS) are generally not required to rate multiple hypothetical health states and intentional framing techniques are not routinely employed. However, a similar unintentional reference type bias may occur due to social comparisons or other life events[11].

Consider a 65 year old woman who is receiving treatment in hospital after suffering a stroke. She may rate her health at this time with reference to surrounding hospital patients who are very unwell. This patient may report her health as 60 out of 100 on the EQ-VAS immediately prior to discharge from an inpatient rehabilitation facility; after considering how much better she is than other patients in very poor health states (near the bottom of the scale). However, immediately after discharge into the care of family, this patient may report her health as 45 out of 100 on the EQ-VAS after considering how much worse her health is in comparison to healthy peers in the community (who may be near the top of the scale). An independent observer may infer that a decline in health state of 15 points has occurred (despite potentially no reduction in the patients' actual health or HRQoL).

Inconsistent consideration of subjective patient reported outcomes may cause a patient to paradoxically report a change when no change has occurred, or a disproportionate change than that which has actually taken place. An inaccurate representation of change due to this type of artefact may have serious implications. In clinical practice this may complicate attempts to evaluate whether a health intervention or disease has resulted in meaningful change in a person's HRQoL. Of no less importance would be the effect that an inaccurate representation of change would have during a randomised trial if all groups were not equally exposed to stimuli prompting a response shift[11]. For example, an intervention group may be required to attend a hospital, clinic or group intervention session resulting in exposure to individuals experiencing extremely poor health states, while a control or comparator group may not be given this same exposure[11].

Despite the previous work by Krabbe and colleagues on multi-item visual analogue scale ratings,[28] there is currently no empirical evidence indicating whether an acute shift in response to a health state scale such as the EQ-VAS may result from a reference type bias when individuals are rating their own health state. The purpose of this study is to illustrate that respondents may not give consistent consideration to the health states that give meaning to the EQ-VAS, and investigate whether merely asking respondents to consider a detailed descriptors of an extremely good health state (Description-A) and extremely bad health state (Description-B) between assessments induces an acute shift in their own EQ-VAS rating. The set of descriptors used as Description A and B are presented in Additional file 1.

It was hypothesized that respondents frequently would not consider what the EQ-VAS scale anchors represent during initial completion of this scale. Furthermore, it was considered likely that many participants would change their overall HRQoL report after consideration of the extreme health descriptors (Additional file 1). It was hypothesized that consideration of extremely poor health descriptors would cause many respondents to increase their reported HRQoL score as they would consider their current health state to be further away from the lower end of the scale, while some would lower their reported HRQoL considering that their current health state was actually closer to lower end of the scale. In the same way after considering descriptors of an extremely good health state many would move their score lower, while some would move their score higher.

It was also considered possible that an order effect may occur whereby patients' responses may be dependent not only on the extreme health state descriptors themselves, but the order in which they were provided. Previous investigations dealing with HRQoL reporting and order effects have generally found no significant order effect[35-38]. However, given the novel nature of this investigation in providing extreme health state descriptors between assessments, this investigation also aimed to examine whether the order in which these descriptors were provided affected the pattern of responses.

## Methods

### Design

**Figure 1:**
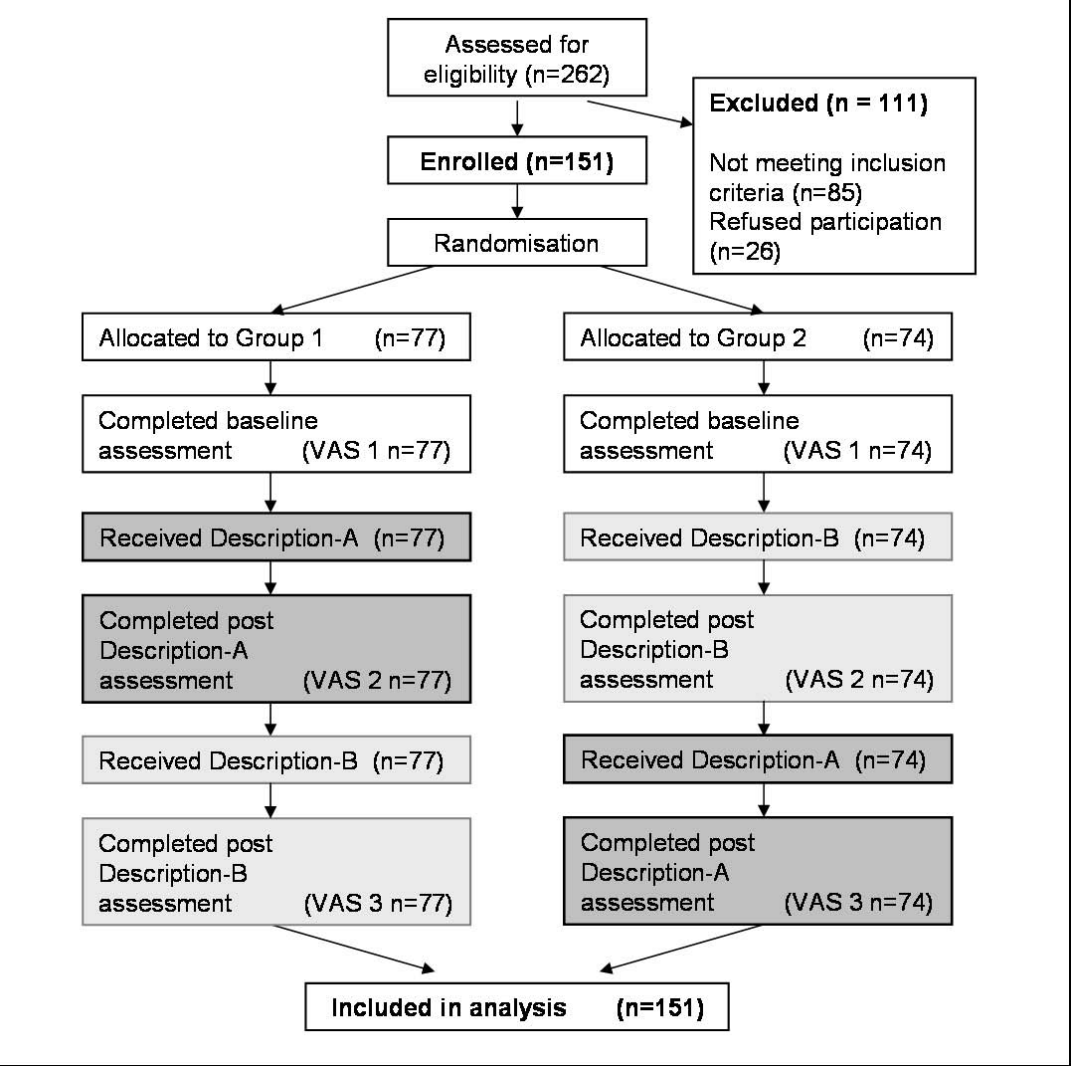
**Study design - Randomised Crossover Trial**.

A two group, randomized crossover design methodology trial was implemented (Figure 1). After completing baseline measurements, patients randomized to group one received Description-A first (this involved being asked to consider the set of good health state descriptors) then Description-B (this involved being asked to consider the set of poor health state descriptors). Patients in group two received Description-B first, then Description-A. There was no washout period between the provision of each of the two health state descriptor sets, as the order effect and effect of receiving both sets of descriptors were under investigation.

### Participants and setting

One hundred and fifty-one patients admitted to the rehabilitation unit of a tertiary hospital in Brisbane, Australia, participated. This population was selected for this investigation for several reasons. The focus of health interventions for this patient group generally focuses on treatments and therapies aiming to maximise function and HRQoL, thus making HRQoL evaluation integral to clinical and research assessments within this type of patient population[3]. This population is also potentially at risk of changing points of reference when completing subjective patient reported outcomes due to social comparisons or life events that have lead them to be in need of hospitalisation[11]. For inclusion in the study patients were required to be able to communicate effectively in English and have basic cognitive functioning intact as indicated by a Mini Mental State Examination (MMSE) score of >23/30[39].

### Measures

The primary outcome measure was the EQ-VAS. This is a continuous measure of overall health state using a 100 point visual analogue scale where 0 represents the worst imaginable health and 100 represents the best imaginable health[18]. This outcome measure was used a total of three times for all participants (Figure 1). The EQ-VAS was first completed at baseline (VAS 1) as a control for comparison purposes, then for a second time (VAS 2) after each group had received their first set of descriptors (Description A or B depended on group). The EQ-VAS was then completed for a third time after the crossover (VAS 3) after each group received the remaining set of descriptors (Description B or A respectively).

As a secondary outcome immediately after responding to the baseline EQ-VAS (VAS 1) before either set of descriptors were provided, participants were asked whether they had "considered what best (and worst) imaginable health may be like." This was recorded as a binary yes/no answer for each anchor. If participants had considered what a best imaginable or worst imaginable health state may be like for either EQ-VAS anchor they were asked to describe in words what they had considered. Their description was recorded verbatim. After receiving each set of descriptors (Description-A or Description-B), patients were also asked if the health state described was more extreme than that which they had previously considered to be the end point on the EQ-VAS (0 or 100 respectively). A dichotomous response to this question (yes/no) was also recorded as secondary outcome measure.

Baseline patient demographics and their Functional Independence Measure score[40] were also collected from the medical record for the purpose of describing the sample.

### Intervention (Description-A and Description-B)

Description-A involved asking the participant to consider a set of descriptors for an extremely good health state (Additional file 1). Description-B involved asking the participant to consider a set of descriptors for an extremely poor health state (Additional file 1). Each set of descriptors required less than one minute to read at a comfortable pace. The descriptors provided to the patient were a compilation of the respective best and worst descriptors for each health component used in the Assessment of Quality of Life (AQoL) instrument[41]. It is noteworthy that both sets of descriptors were not intended to affect the patients underlying health, and thus were health evaluation methodology interventions rather than intended as any kind of clinical intervention. The descriptors were intended to promote more careful consideration of a range of possible HRQoL attributes by the respondent immediately prior to assigning an EQ-VAS value to their own health state.

### Procedure

Ward staff identified potential participants who were then approached by a research assistant (RA1). RA1 explained the study and sought informed written consent. RA1 was not aware of the randomisation sequence (calculated using computerised random number generation by a blinded member of the investigative team and stored in a locked filing cabinet). Consenting participants were then allocated to group (one or two) in order of the random sequence according to their participant number by a separate research assistant (RA2). Before receiving either set of descriptors, patients in both groups completed a baseline self-report of the EQ-5D questionnaire including the EQ-VAS (VAS 1), and the relevant secondary outcomes.

Group one received the health state descriptor sets in the alternative order to group two (Figure 1). After receiving being asked to consider the first set of health state descriptors (Description A or B depending on group), participants completed the assessment measures which included a second self-report of the EQ-VAS (VAS 2) and the secondary outcome measures. Once participants had completed these assessment measures the remaining set of health state descriptors (Description B or A respectively) was immediately given and patients then completed a third and final self-report of the EQ-VAS (VAS 3) and the relevant secondary outcomes.

The assessments and health state descriptors were administered in this way, only minutes apart, to eliminate the possibility of an actual change in underlying health state. This investigation was approved by the Princess Alexandra Hospital and The University of Queensland's Human Research Ethics Committees.

### Power analysis

When examining the main effect comparison of Description-A versus Description-B on EQ-VAS scores after each set of descriptors, this experiment had 90% power to detect a conservative between-groups difference in VAS of 3 points assuming a standard deviation of 17.5 using total sample size of 150 and a two tailed alpha of 0.05. Because of the correlation of responses within patients, this sample size had >90% power to detect a similar change in VAS when examining the within-group main effect of providing both sets of descriptors between baseline (VAS 1) and the final follow-up assessment (VAS 3).

### Data Analysis

Demographic and baseline EQ-VAS data were tabulated (Table 1). Raw data was checked for normality graphically and using tests for skew and kurtosis[42,43]. Difference between groups in baseline EQ-VAS score (VAS 1) was examined using an unpaired t-test. Three change scores for the EQ-VAS were calculated. These were the difference between the baseline EQ-VAS and the EQ-VAS completed after receiving the first set of descriptors (VAS 2 -VAS 1), the difference between EQ-VAS after the first set of descriptors and the final EQ-VAS after the second set of descriptors (VAS 3 -VAS 2) and the difference between the baseline EQ-VAS and the final VAS after the second set of descriptors (VAS 3 -VAS 1).

**Table 1:** Participant Demographics, baseline EQ-VAS and Functional Independence Measure scores

	Group 1 n = 77	Group 2 n = 74
Age - median (IQR)	80 (74-86)	79 (73-86)
Mini Mental State Examination - median (IQR)	27 (25-29)	26 (25-29)
Diagnosis category		
Stroke	7 (9%)	9 (12%)
Other Neurological	2 (3%)	3 (4%)
Orthopedic (non elective)	23 (30%)	20 (27%)
Orthopedic (elective)	1 (1%)	2 (3%)
Other Musculo-skeletal	2 (3%)	2 (3%)
Cardiac	3 (4%)	3 (4%)
Pulmonary	8 (10%)	5 (7%)
lower limb amputation	17 (22%)	16 (22%)
Other Medical Condition	8 (10%)	8 (11%)
Other Geriatric Condition	7 (9%)	6 (8%)
Functional Independence Measure		
Cognition - median (IQR)	33 (31-34)	32 (30-33)
Motor - median (IQR)	61 (44-68)	59 (47-70)
Baseline EQ-VAS - mean (SD)	59 (19)	56 (16)

The number (and percentage) of respondents who changed their EQ-VAS by 5 points or more (in either direction) after exposure to the good and poor health state descriptors was calculated (Table 2). These calculations were done in order to evaluate the effect of the health state descriptors at an individual level (as opposed to group mean differences). This analysis was considered important as analysis of group means would only reflect a systematic change (i.e. a general increase or a general decrease in EQ-VAS scores). However, some individuals may have reported positive shifts while others report negative shifts (depending on their response to the health state descriptors). If shifts in response occurred in a less uniform way such as this, these changes may cancel one another out resulting in no significant mean change. Such a finding may mask response shifts that may have been interpreted as meaningful change in a clinical setting where decisions are likely to be based on an individual patient's reported change. This is in contrast to changes in group means which are more likely to affect the interpretation of clinical trial findings. To investigate mean EQ-VAS changes two mixed 2x2 ANOVAs were also conducted.

**Table 2:** Number of participants who increased or decreased their EQ-VAS self report by 5 points or more after exposure to either good or poor health state descriptors as well as after both sets of descriptors.

	Group 1 n = 77			Group 2 n = 74		
	Number (%) Increase ≥5 points	Number (%) Decrease ≥5 points	Number (%) Either direction ≥5points	Number (%) Increase ≥5 points	Number (%) Decrease ≥5 points	Number (%) Either direction ≥5points
Good health descriptors (Description-A)	29 (38%)	24 (31%)	53 (69%)	11 (15%)	25 (34%)	36 (49%)
Poor health descriptors (Description-B)	37 (48%)	14 (18%)	51 (66%)	45 (61%)	8 (11%)	53 (72%)
After both Description A and B (compared with baseline EQ-VAS)	39 (51%)	12 (16%)	51 (66%)	40 (54%)	15 (20%)	55 (74%)

The first ANOVA investigated whether providing the good health descriptors had a different effect than providing the poor health descriptors and whether this was dependent on the order in which the descriptors were provided. To examine this, the first ANOVA investigated the main effects of Description (A versus B) and sequence (i.e. whether participants were in the group who received best or worst health descriptors first), and an interaction effect between them. This analysis examined the change between the EQ-VAS rating taken after respondents were exposed to each set of health state descriptors (after Description A or B) and the EQ-VAS rating taken immediately prior to the provision of that set of descriptors.

The second ANOVA investigated whether the final EQ-VAS rating after the provision of both good and poor health state descriptors (VAS 3) was different to the baseline EQ-VAS report (VAS 1) and whether this was dependent on the order in which the descriptors were provided. To examine this, the second ANOVA investigated the main effects of total change in HRQoL (VAS 3 -VAS 1) and sequence (i.e. group), and the interaction between total change in HRQoL and sequence (i.e. group).

## Results

One hundred and fifty-one patients were enrolled in the study. All participants completed each assessment and were included in analysis. The groups' baseline demographics were comparable (Table 1) with no mean difference in baseline EQ-VAS between groups (p = 0.30).

Immediately after completing their baseline EQ-VAS, 74 (49%) participants reported that they had not considered what best imaginable health (top scale anchor) may be like and 85 (66%) had not considered what worst imaginable health (bottom scale anchor) may be like. Of those participants who did think of a best imaginable health state, 59 (77%) thought the set of good health descriptors (Description-A) was more extreme (better) than the health state they had previously considered as the top scale anchor. Of those participants who did think of a worst imaginable health state, 63 (95%) thought the set of poor health descriptors (Description-B) were more extreme (worse) than the health state they had previously considered as the bottom scale anchor.

The number of participants in each group who changed their EQ-VAS report by 5 points or more after exposure to each of the health state descriptors are presented in Table 2. The majority of patients in both groups either increased or decreased their VAS score after being exposed to the good and poor health state descriptors. When comparing the final EQ-VAS score after both sets of health descriptors had been provided (VAS 3), to their baseline score (VAS 1) 106 (70%) of all participants had a final health VAS self-report that differed by 5 points or more from their baseline VAS; 51 were from group one and 55 were from group two.

**Figure 2:**
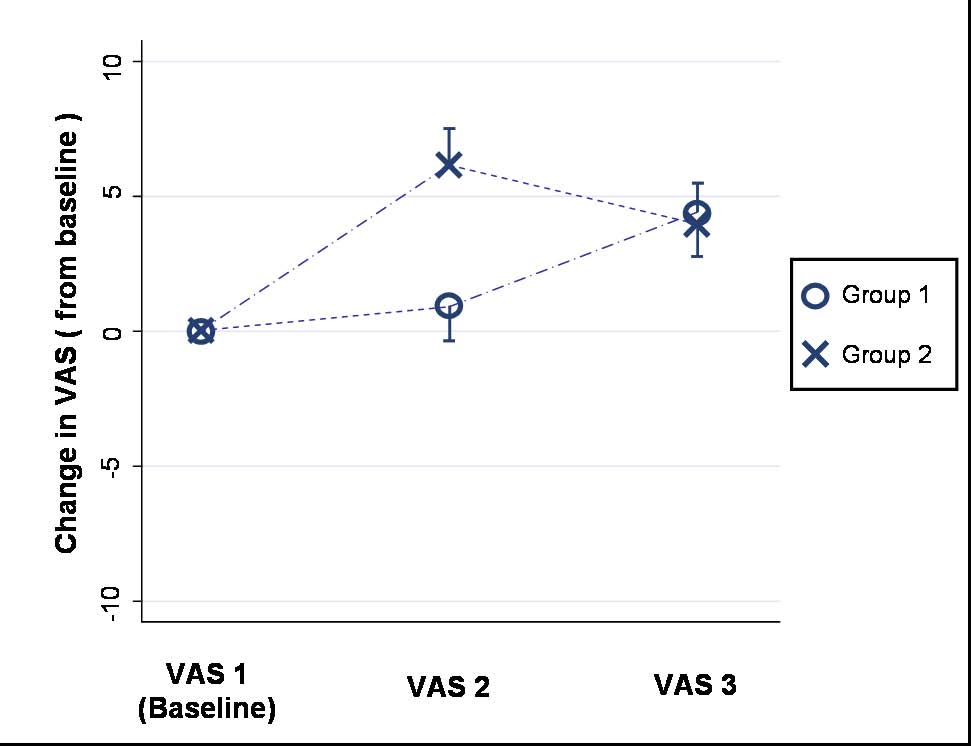
**Mean difference (and standard error) from baseline at each assessment by group**.

The first ANOVA investigating whether providing the good health descriptors had a different effect than providing the poor health descriptors revealed this main effect of Description (A versus B) was significant (df = 1,149; F = 11.88; p < 0.001). A slight difference between groups in response to the good health descriptors observed in Figure 2 (slight increase for group one, small decrease for group two) was not significant with the main effect of sequence (df = 1,149; F = 0.24, p = 0.623) and the interaction (df = 1,149; F = 0.07, p = 0.793) both non-significant. Data from both groups combined indicated that the poor health descriptor set caused a mean (SD) increase in VAS score of 4.88 (11.81) points while the good health descriptor set caused a mean (SD) decrease in VAS score of 0.35 (10.71) points when compared with the VAS score immediately prior to that set of descriptors.

The second ANOVA which investigated the main effect of mean change in EQ-VAS after exposure to both sets of descriptors (VAS 3 -VAS 1), revealed that both groups' final mean EQ-VAS score was higher than their baseline EQ-VAS score (df = 1,149; F = 21.21; p < 0.001). The order in which the descriptors were received was non-significant with the main effect of sequence (df = 1,149; F = 2.11 p = 0.148) and the interaction effect (df = 1,149; F = 0.13 p = 0.723) both non-significant. The overall data from both groups combined indicated a mean (SD) difference between the final EQ-VAS (VAS 3) and the baseline EQ-VAS (VAS 1) for all participants was 4.5 (12.0) points, VAS 3 was higher. This is also illustrated in Figure 2 where no substantial difference between the mean change scores from each group at the final assessment point (VAS 3) existed.

## Discussion

### Overall Outcome

The findings from this investigation support our hypothesis that respondents frequently do not give consistent consideration to the health states which give meaning to a health state scale such as the EQ-VAS. This may have a substantial effect on how a respondent reports their HRQoL on rating scales of this nature. This investigation has been the first to demonstrate that patients' self-report of their own HRQoL can be substantially altered despite no actual change in their underlying health state occurring (Table 2 and Figure 1). A change in self reported EQ-VAS rating was elicited for a large proportion of individuals merely by asking respondents to consider a set of health state descriptors (Table 2).

As one would expect, the mean baseline EQ-VAS score (VAS 1) for this hospitalised patient sample was substantially lower than the previously reported population norm of 82.5 out of 100[44]. Despite anchors of best imaginable and worst imaginable health state being present in the standard application of this instrument, participants frequently did not consider what these anchors might represent. Overall 133/151 (88%) and 148/151 (98%) of participants either reported that the descriptors of very good and very bad health states (respectively) were more extreme than they had previously considered for the respective end anchor points or that they had not considered best and worst imaginable health states at all during standard completion of the EQ-VAS.

Overall 70% of participants changed their self-report of HRQoL on the 100 point scale by a margin of 5 points or more after being provided with detailed descriptors of both good and poor health states (Table 2). These changes were not uniform across individuals, with 79 (52%) increasing and 27 (18%) decreasing their EQ-VAS rating by 5 points or more.

At the present time there is no available, published value for minimal clinically important difference on the EQ-VAS amongst this type of population. However a change of this magnitude is comparable to what has previously been identified as clinically important change on this scale amongst other patient populations[45-49]. Furthermore in the context of this population, a change of 5 points or greater represented a change of 8.5% or greater of the mean baseline score. Thus this amount of change in self-reported HRQoL on this scale may well have been interpreted as clinically meaningful for up to 70% of participants despite it being attributable to an acute shift in response rather than a change in underlying health. If this were observed in a clinical setting, these reports may have incorrectly been interpreted as improvement in HRQoL for individuals who increased their score, and as decline in HRQoL amongst those who decreased their score (Table 2).

While it is unlikely that a patient will come across extreme health state descriptors between health assessments unless they are provided to them explicitly, other naturally occurring events (such as exposure to patients in an extremely poor health state while attending a hospital, watching television or elsewhere in the community) are likely to affect how a respondent completes a self evaluation of their own health state.

### Strengths and limitations

A strength of this investigation lies in the methodology of employing a randomised crossover trial design for this novel examination of HRQoL evaluation. This has allowed for a methodologically rigorous investigation resulting in empirical evidence to support our hypothesis. This proof of concept is likely to contribute to future improvement in self-reported health evaluation methodology relevant to clinical settings, epidemiological investigations and health research utilising patient reported outcomes. However, the ability to directly generalise these results is limited by the population in this study being hospitalised older adults and the use of a single rating scale (EQ-VAS) as the primary outcome. It is possible that other populations and rating scales may have been affected to a greater or lesser extent. However, given the high use of healthcare resources by this population and the widespread use of the EQ-5D instrument, the sample and EQ-VAS were appropriate for this investigation.

### Comparison to prior research

The metric properties and theoretical basis of visual analogue rating scales for use in evaluating health states has been the subject of much investigation and debate[11,28,29,50-58]. Previous empirical work has demonstrated that EQ-VAS ratings can be dependent on the context in which they are presented when rating multiple hypothetical scenarios[28]. While that finding has important implications regarding the use of multi-item visual analogue scales for assigning utility values to hypothetical health states,[28] this investigation has been the first to highlight the risk of a reference type bias on influencing individuals report of their own HRQoL using a rating scale such as the EQ-VAS.

The novel nature of this investigation limits the direct comparisons that can be made to previous empirical investigations of the response shift phenomenon. Research investigations in the response shift field have often focused on analysis of mean scores or changes at a group level [59-62] as opposed to changes at an individual level[8,17,63]. While this investigation found significant effects at a group level with changes in mean EQ-VAS ratings, non-uniform response shifts across a large proportion of individuals were also observed (Table 2).

Findings from this study are consistent with previous investigations of social comparison, framing and order effects. It has previously been identified that self-reports of quality of life and HRQoL are dependent on social comparisons[64-67]. It is likely that the descriptions of good and poor health states presented in this investigation may have elicited a similar effect to previously described upward or downward social comparisons respectively[64,66,67]. The resultant change in EQ-VAS that occurred after this stimuli is also congruent with investigations of the framing effect[30-33]. While the current investigation did not alter the wording of the EQ-VAS to give a positive or negative valence, a similar effect is likely to have been elicited by the extreme health state descriptors provided between assessments. Interestingly, the order (sequence) in which the descriptors were provided in this investigation was not statistically significant. This is consistent with previous investigations that have revealed the order of instrument administration to be inconsequential[35-38,68].

### Implications and future directions

The EQ-VAS instrument was used in this investigation to illustrate how variable consideration during the evaluation process can cause substantially different reports of HRQoL, despite no actual change in underlying health. Rather than an indictment of this particular instrument (which is certainly not the intention of the authors), these results indicate that caution should be exercised when using subjective patient reported outcomes such as those dependent on extreme anchors to give meaning to the value assigned to an individual health state.

It is clear from the minimal amount of consideration of the anchors by the respondents during the standard administration of the EQ-VAS, and their desire to change their response after being asked to consider the health state descriptors in this study, that responses are frequently not well considered. It is possible that many respondents may have initially applied an unwritten qualifying context for the anchors, such as best or worst health 'that is possible for me,' 'that I have experienced,' 'for my age', or some other social comparator. Further investigation of what the respondents considered would be useful to support or refute this speculation. Empirical evidence of this nature would be useful to inform future improvements in HRQoL evaluation methodology. This empirical evidence could be generated through qualitative analysis of a direct think aloud approach or probing questions immediately following standard completion of the instrument[69].

Based on findings from this investigation it may be possible to promote consistent consideration of HRQoL scales by artificially creating a standardised frame of reference for an instrument. In the case of the EQ-VAS respondents may be asked to consider a broad description of an extremely good and poor health state, like those used in this study, before completing the EQ-VAS. We are not suggesting that these health descriptors represent best and worst imaginable health. Rather, they may act as stimulus for respondents to consider a spectrum of health components, and give reasonable consideration to how extreme health states can be. If this occurred at each assessment, it may promote consistent consideration of the instrument.

Considering the spectrum of health components included in the health state descriptors may potentially reduce reconceptualisation and reprioritisation, while considering the extreme nature of how bad (or good) each of the health components can be may help reduce recalibration. Further investigation in this area is warranted, and would most likely require use of custom designed evaluation measures or approaches. Further research is also indicated to determine if extreme health states which give meaning to health rating scales are frequently not considered amongst other patient populations. Investigation of the issues addressed in this manuscript should also be examined amongst other patient reported outcomes including pain and fatigue.

## Conclusions

Subjective health state evaluations may not be well considered. An immediate significant shift in response can be elicited by exposure to a mere description of an extreme health state despite no actual change in underlying health state occurring. Caution should be exercised when interpreting change in subjective patient reported outcomes in research and clinical settings; particularly those dependent on brief extreme anchors to give meaning to assigned values.

## Competing interests

The authors declare that they have no competing interests.

## Authors' contributions

All authors contributed to the conception of research idea and planning of research processes. SM (and research assistants) contributed to data collection. SM and TH contributed to data analysis. SM prepared the manuscript. All authors contributed to manuscript review, appraisal and editing.

## Supplementary Material

Additional file 1 **Health state descriptors**. This file contains the health state descriptors used for Description-A and Description-B. Click here for file
