# Supplementary material for: Reference bias: presentation of extreme health states prior to eq-vas improves health-related quality of life scores. a randomised cross-over trial
Source: Health Qual Life Outcomes. 2010 Dec 2;8:146. doi: 10.1186/1477-7525-8-146 (PMC3014890; doi:10.1186/1477-7525-8-146)
Supplement: Additional file 1 — Health state descriptors. This file contains the health state descriptors used for Description-A and Description-B. [file 1477-7525-8-146-S1.DOC]

**Description-A**

A person in this health state does not need any medication or require any medical treatment. They do not require any help with personal care or household tasks. They have no difficulty getting around in their own home or in the community. Their relationships with friends, partner or family are close and warm and not affected by their health. They are never socially isolated or lonely. Their role in their family is not affected by their health. They have normal vision and hearing. They have no difficulty communicating with others. The have no difficulty sleeping at night. They are not anxious, worried or depressed. They have no pain at all.

**Description-B**

A person in this health state relies on taking many medications. Their life is dependent upon regular medical treatment. They need daily help with all personal care and household tasks. They are unable to get around in the community or even their own home by themself. Their poor health has resulted in no close or warm relationships with friends, partners or family. They are socially isolated and lonely. They are not able to carry out any part of their family role. They have no vision (blind) or hearing (deaf). They are unable to adequately communicate with others. They are only able to sleep in short bursts and are awake most of the night. They are extremely anxious, worried and depressed. They also suffer unbearable pain.
